# The BIOMES℠ Tool: An Approach to Recognizing Wound Severity for Early Intervention and Referral to a Specialist

**DOI:** 10.7759/cureus.89352

**Published:** 2025-08-04

**Authors:** Trent Brookshier, Laura Swoboda, Chrystalbelle Rogers

**Affiliations:** 1 Podiatry, North Park Podiatry, San Diego, USA; 2 Translational Science, Carthage College, Kenosha, USA; 3 Clinical Services, Hartmann USA, Rock Hill, USA

**Keywords:** diabetes, peripheral arterial disease, referral criteria, screening tool, wound healing

## Abstract

The escalating prevalence and incidence of individuals with chronic, non-healing, or hard-to-heal wounds is staggering and poses significant financial burdens on the healthcare system. Initial wound evaluations and assessments are often performed first by urgent care, emergency responders, or even general medicine professionals who are skilled practitioners but are not chronic wound specialists and may have only received a limited amount of education or training specific to wound care. As a result, these medical providers may not be familiar with current wound care scoring systems that aid in determining wound severity, guiding early interventions, and identifying the need for specialized care based on patients’ overall medical condition and wound status. A referral to a wound care specialist has the potential to expedite healing, reduce the overall cost of care, alleviate patient suffering, and ultimately, save a limb or a life.

The blood flow, infection, offloading, metabolic issues, and exudate, social (BIOMES℠) tool is a coined acronym that can be used by any healthcare provider to quickly identify whether a patient should be referred to a specialist by classifying a wound as low, moderate, or high risk for delayed healing. Blood flow, infection/bioburden, offloading/overloading, metabolic/morbidity, exudate/edema, and social/economic barriers are assessed, and 1 point is assigned to each barrier that can be identified as a red flag with the potential to affect the healing trajectory of a patient’s wound. Wounds are classified as follows: low risk: no barriers; moderate risk: 1 BIOMES℠ barrier; high risk: 2 or more BIOMES℠ barriers. Any patient identified as moderate or high risk should be referred to a wound care specialist in hopes that earlier, more aggressive wound care and medical management will result in improved wound healing versus traditional wound management. Addressing these barriers is essential for wound management and improved outcomes, especially in lower extremity care.

## Introduction

Significant shifts in chronic wound care prevalence and cost of care have been reported over recent years, including increased costs incurred in the physician’s office and decreased costs in hospital-based outpatient wound care clinics [1]. An estimated 16.3% of Medicare beneficiaries have a chronic or hard-to-heal wound with a total cost of care exceeding $22 billion. Between 2014 and 2019, fees for wound care provided in physicians’ offices increased from $3.0 billion to $4.1 billion [1]. Numerous studies have shown that early intervention, including proper diagnosis, adequate wound bed preparation, and use of advanced strategies to facilitate wound healing, leads to faster wound healing and reduced cost of care [2-6]. A retrospective study of patients with hard-to-heal lower extremity wounds who were referred to a multidisciplinary wound care service found that the patients had better healing rates with reduced costs and improved patient quality of life as compared to when they were treated in non-specialized settings [7]. A study by Takahara et al. found that patients with critical limb-threatening ischemia often had their wounds present for an average of one to three months before referral to a vascular specialist [8]. A review of guidelines for primary caregivers treating wounds revealed delayed referrals and, in some cases, treatments that led to worsening outcomes, including elevation for an arterial wound that is not making progress [9]. In addition, offices of primary care providers often do not stock the supplies, advanced dressings, and equipment necessary for best practice wound care.

Current literature emphasizes the importance of not just managing a wound but treating a patient who has a wound with the intention of wound healing by using best practice strategies based on the underlying pathology and wound diagnosis, as well as optimal management of any comorbidities the patient may have [10,11]. Indeed, signs that a wound may not be likely to heal in a timely manner may be identified within 24 hours of wound onset by recognizing the red flags. Some signs indicate that the healing process is likely to stall during the inflammatory phase [10]. Other signs predict whether or not the patient will have adequate dressings or supplies necessary to help with offloading or drainage of certain types of wounds. 

It is critical to proactively identify, as early as possible, any red flags that may indicate or trigger stalled healing rather than waiting the oft-cited 12 weeks for a wound to be deemed chronic; the longer a wound remains in the stalled healing phase, the harder it is to effectively heal. Worse still, the longer the patient has a wound, the higher the chance it will lead to a limb-threatening amputation or a life-threatening septic event. Visible red flags include a lack of angiogenesis and granulation tissue formation, aberrant local inflammation, signs of infection, rolled edges, presence of high exudate, or signs of local tissue infection. These wounds can be categorized as high risk, indicating a need for comprehensive care to ensure that underlying factors that impede wound healing are addressed, barriers to healing are removed, and healing potential is optimized [12].

The BIOMES℠ (blood flow, infection, offloading, metabolic issues, exudate, social) tool can assist any healthcare provider who identifies a wound and can influence a patient’s wound healing trajectory, as well as the barriers to healing that may require specialized care. Following application of the BIOMES℠ tool, a wound is classified as low, moderate, or high risk for non-healing. If the wound is determined to be moderate to high risk, early referral to a wound care specialist and other supporting medical disciplines (e.g., podiatrists, endocrinologists, vascular specialists, physical therapists, nutritionists) can result in shorter healing times, reduced costs of care, and improved patient quality of life. The resources provided by a wound specialist who has access to advanced therapies and specialized training in treating wounds, in conjunction with the care provided by the primary physician, create an environment of “wound balance,” shifting the focus from managing wounds to leveraging the clinical intention of healing wounds as early as possible.

## Technical report

Definition of the BIOMES℠ tool

All dermal wounds have areas where the skin has become compromised or lost, thus allowing the microbiota (fungi, bacteria, and viruses) that are on the skin and in the environment to freely invade the tissue. The host’s normal protective mechanisms and immune responses to injury are affected, and the surrounding tissue is at risk for developing infection. 

In addition to infection, there are other key elements that can influence a wound’s ability to heal, and if these barriers to healing are identified and addressed early in treatment, healing can occur in a shorter length of time, reducing both patient suffering and the cost of care.

Current best practices for wound care focus on healing wounds as early as possible, which includes making a correct diagnosis, recognizing the red flags that impede wound healing, and initiating early intervention rather than waiting 12 weeks to deem a wound as chronic [13].

Wound chronicity is not time-dependent; rather, it is based on wound characteristics, and recognizing early signs of risk for wounds to become stalled or non-healing has become increasingly important. The BIOMES℠ scoring scale is a tool that accounts for the elements affecting the wound’s ability to heal and the wound characteristics affecting wound chronicity. The BIOMES^SM^ scoring scale serves as a swift and effective method to quickly assess wounds as moderate- to high-risk for being hard to heal. 

The BIOMES℠ acronym provides the clinician performing initial patient assessments with succinct direction in the early identification of barriers to healing of moderate- to high-risk wounds that can potentially lead to decreased quality of life, loss of limb(s), or even loss of life. Each letter guides the clinician to evaluate a specific aspect of the patient’s medical history or wound presentation that may affect their healing trajectory.

BIOMES℠ barrier descriptions

Blood Flow

Peripheral arterial disease (PAD) is the most common cause of insufficient blood flow to the lower extremities and can either cause a wound or impede the healing of wounds caused by other etiologies. Wounds caused by PAD have a distinctive location and appearance (Table 1). As with all wounds on the lower extremities, vascular assessment is a critical component of patient evaluation and includes pulse palpation, capillary refill, rubor of dependency, and ankle-brachial index (ABI) if the equipment is available. Any patient with diminished or absent pulses, dermatologic signs of PAD, or an ABI less than 0.8 should be referred to a vascular specialist [14]. An ABI greater than 1.2 is consistent with the noncompressible vessels in patients with diabetes, in which case, toe pressures are measured [15]. These patients should also be referred to a vascular specialist.

**Table 1:** Characteristics of the four typical wound etiologies PAD: peripheral arterial disease. Developed by Rose Hamm and Trent Brookshier, DPM, used with permission.

WOUND EVALUATION BY ETIOLOGY					
	Location	Tissue	Pain	Skin	Exudate
Arterial	Malleolus and feet, distal digits (toes or fingers)	Dry, necrotic or slough, little or no granulation. Tendons or ligaments may be exposed	Yes. May have intermittent claudication, dependent leg syndrome, or rest pain	Dry, hairless, shiny, thin, positive rubor of dependency	None unless infected
Venous	Lower 1/3 of the leg (called the gaiter area)	Red or pink, poorly defined edges, yellow slough. Poor granulation	Generally, not painful except for vasculitis or infection	Hemosiderosis (dark, brawny appearance). Atrophie blanche. Lipodermatosclerosis. Peau d’orange texture	Variable, moderate/heavy, serous drainage
Pressure	Over bony prominences	Varies from non-blanchable erythema, to dark red or maroon color, to eschar. Fascia, muscle, tendon, or bone if full-thickness	Varies depending on the structures involved	Discolored, erythematous, or hypoxic May be macerated or excoriated	Varies
Diabetic	Interdigital spaces or any portion of the foot coming in contact with ground or shoegear	Callus or blister, slough, may probe to bone, necrotic with PAD	Often none. May indicate infection. Neuropathic pain may vary [16]	Dry, thick, scaly, hyperkeratotic	Varies, depending on infection

Conditions other than PAD may cause ischemic wounds that often mimic arterial wounds but require more specialized treatment and include the following: arterial entrapment, thrombus, adventitial cyst, embolism, fibromuscular dysplasia, trauma, vasculitis, vasospasm (Buerger disease or thromboangiitis obliterans), and cutaneous lymphoma. Cardiac disease, sedentary lifestyle, and history of smoking or vaping may also contribute to decreased blood supply to a distal extremity wound with poor healing potential. Treatment of the underlying disorder and restoration of blood flow are paramount to successful wound healing.

Venous wounds are the most common type of lower extremity wound in the elderly population and have the typical appearance described in Table 1, as well as some degree of lower extremity edema. An estimated 10-18% of patients with a venous wound have some degree of PAD [17]; thus, a comprehensive vascular screening is advised for all patients with venous wounds, as well as referral to a vascular specialist to monitor the progression of the PAD.

Infection/Bioburden

All wounds have flora on their surfaces, and their effect on wound healing and the need for antimicrobial therapy depends on the number of microbes, the type of microbe (bacterial, viral, or fungal), and the host immune system [18].

Local infection (previously termed critical colonization) usually requires advanced antimicrobial dressings. Deeper infections or more aggressive cellulitis may require systemic antibiotics. Both are best treated by wound care and/or infectious disease specialists. If necrotizing fasciitis is suspected, the patient should be referred immediately to a hospital for emergency surgery and antibiotic therapy, which is crucial for limb preservation and patient survival. Early local signs of necrotizing fasciitis are at times difficult to detect because they can present similarly to other non-life-threatening chronic wounds with characteristics including edema, induration, erythema, and blistering, and a history of minor skin trauma. Systemic signs include fever, pain, tachycardia, and elevated white blood cell count. These patients cannot be successfully treated in outpatient facilities; they require immediate debridement of the infected tissue by surgical procedures, along with intravenous antibiotics.

Biofilms on a wound surface are diverse communities of bacteria embedded within a self-produced matrix of an extracellular polymeric substance that is resistant to topical antimicrobial therapy [19]. Removal of the biofilm is imperative for wound healing to progress and requires debridement skills, advanced dressing knowledge, and consistent therapy, all best provided by a wound care specialist [20-22].

Offloading/Overloading

Overloading, or high peak pressure in vulnerable areas of a patient’s body, is a critical issue for two different populations: patients with neuropathy and patients at risk for pressure injury. Those with diabetes typically have neuropathy and thus cannot feel when the wound has pressure or repeated trauma that results in tissue necrosis. Comprehensive screening of a patient with diabetes includes foot inspection for deformities caused by motor neuropathy, sensory testing for inability to feel pressure, and vascular screening for PAD, which is a common complication among these patients [23-26]. Foot deformities create high peak pressures on the plantar surface of the foot during standing and gait, especially the metatarsal heads, which then cause shear between the bone and soft tissue, resulting in diabetic foot ulcers (DFU) (Table 1). Pressure redistribution, or offloading, is the most important treatment strategy for DFUs (e.g., with total contact casts or removable cast walkers) and should be provided by an experienced wound care specialist, such as a wound and ostomy care nurse or podiatrist whose expertise includes diabetic feet. In some cases, surgical reconstruction is required to remove bony prominences or correct a deformity causing the wound or keeping the wound present [27,28].

Patients with or at risk for pressure injuries require comprehensive care, including rehabilitation services to optimize mobilization, support surfaces to redistribute pressure from the affected tissues, prophylactic dressings for bony prominences, nutritional support, and, in some cases, plastic surgery for wound closure. The National Pressure Injury Advisory Panel has clearly defined the stages of pressure injury and advises that care must be provided by a trained multidisciplinary team [29]. When screening a patient with dark skin for pressure injuries, changes in skin color may not be visible or blanch with pressure, in which case skin temperature, texture, and/or hyperpigmentation should be monitored [30,31]. Meticulous skin assessments are necessary, and referrals are needed when overloading is identified as a risk factor and special support surfaces or offloading devices are indicated. 

Metabolic/Morbidities

Diabetes is the most common comorbidity seen in patients with hard-to-heal wounds [32-34]; however, there are other conditions that can impede wound healing, such as end-stage renal disease or peripheral vascular disease. These conditions must be identified and managed for healing to occur. Autoimmune disorders such as systemic lupus erythematosus, pyoderma gangrenosum, pemphigoid, hidradenitis suppurativa, and drug-induced hypersensitivity syndrome can cause wounds [35] with atypical characteristics (as compared to the four typical wound etiologies) that alert the clinician to an unusual diagnosis requiring the care of medical and wound specialists (Table 2). In addition, the medications used to treat many comorbidities may have a negative effect on wound healing, e.g., steroids, nonsteroidal anti-inflammatory drugs, anti-rejection medications, chemotherapy, and anticoagulants [36,37]. Discontinuing or reducing the dosage of medications is a decision that the prescribing physician and patient must make together by weighing the consequences of comorbidity symptoms returning against the impact on wound healing.

**Table 2:** Characteristics of an Atypical Wound Developed by Trent Brookshier, DPM, Laura Swoboda, DNP, and Chrystalbelle Rogers, MSN.

Characteristics
Unusual location, e.g., calf, thigh, abdomen
Unusual age of the patient, e.g., a necrotic wound on the toe of a young person
Asymmetrical lesion shape
Granulation that extends over the wound edge
Exuberant granulation or callus
Friable granulation tissue, as seen on either malignant or infected wounds
Purple-red border (termed violaceous), suggestive of pyoderma gangrenosum
Ulcer in the center of pigmented lesion
History of repeated trauma or a burn (possible squamous cell carcinoma)
Rolled out edges (suggestive of combined arterial/venous insufficiency if on the lower leg)
Fungating growth (suggestive of malignancy)
History of radiation therapy
No obvious diagnosis with failure to respond to standard care

Regardless of the wound etiology, patients with diabetes must have adequate blood glucose control for healing to occur. The American Diabetes Association recommends that hemoglobin A1C be less than 7% and the fasting plasma glucose be 90-130 mg/dL [38]. Glycosuria begins at a blood glucose level above 180 mg/dL, resulting in dehydration. Also, neutrophil function becomes impaired, worsening infection and wound healing outcomes. Both diabetic educators and registered dietitians are recommended to help patients with diabetes understand the importance of blood glucose control and adopt strategies to meet their goals.

Protein-energy malnutrition impedes wound healing and is detected by the following six patient characteristics: insufficient energy intake, weight loss, loss of muscle mass, loss of subcutaneous fat, localized or generalized fluid accumulation that may mask weight loss, and diminished functional status as measured by hand-grip strength [39].

Obesity can also affect a patient’s healing potential, especially if the wound is in tissue with large amounts of adipose, which has less arterial supply than other tissues and is more prone to infection [40,41].

Exudate/Edema

Wound exudate can be serous, sanguineous, purulent, or any mixture of the three and can indicate the healing phase of the wound. Exudate can also be a sign of infection, venous insufficiency, lymphedema, or inflammation. Although production of wound exudate is a necessary part of the healing process, exudate can adversely affect wound healing when in the wrong amount, in the wrong place, or of the wrong composition [42]. Wound exudate has also been shown to have elevated levels of MMPs, which contribute to delayed healing [43]. Both the type and amount of exudate are critical to the care plan for healing a wound.

Using appropriate absorbent dressings (Table 3) can reduce the frequency of dressing changes and thereby reduce the cost of care. The application of superabsorbent polymer-containing (SAP) dressings helps reduce the expression of some pro-inflammatory proteins (e.g., matrix metalloproteinases (MMPs) and neutrophil elastase) in chronic wounds, minimize the local inflammation, and promote the shift from stagnated healing to healing progress [44]. These dressings are not typically available in a medical clinic; therefore, treatment is best provided by a wound care specialist who can make decisions on appropriate dressings in a setting where such dressings are available [45,46].

**Table 3:** Considerations for wound dressing selection MMPs: matrix metalloproteinases; MVTR: moisture vapor transmission rate; NPWT: negative pressure wound therapy; SAP: superabsorbent polymer. Developed by Trent Brookshier, DPM, Laura Swoboda, DNP, and Chrystalbelle Rogers, MSN.

Dressing Type	Benefits	Shortcomings/Considerations	Wound Balance Alignment
Superabsorbent Dressings (SAP) (especially multilayer silicone border SAP)	High absorption and retention capacity	Not ideal for dry wounds	Excellent for early intervention
	Sequestration of proteases (e.g., MMPs), bacteria, inflammatory cytokines		Aligns with protease modulation goals
	Maintains moist wound environment		Supports biomarker shift and wound balance
	Silicone border for atraumatic removal		
	MVTR enhances wear time and fluid handling		
	Clinically shown to normalize biomarkers in 14 days [44]		
	Maintains fluid retention capacity under compression		
Alginates and Gelling Fiber Dressings	Absorbs moderate to heavy exudate	May cause bleeding during dressing changes	Moderate—Useful for autolysis but can be problematic in bleeding-prone wounds or those with fragile vessels
	Forms a gel, supporting autolytic debridement	Requires secondary dressing	
	Promotes granulation tissue growth	Not ideal for low exudate wounds or dry wounds	
	Some variants have hemostatic properties (via calcium ions)		
Foam Dressings	Absorbs moderate exudate	Limited capacity for heavy exudate	Limited—Useful in stable, moderate-exudate wounds but does not actively modulate chronicity-driving factors
	Cushions and protects wound	Limited ability to sequester/retain proteases or bacteria	
	Comfortable, conformable	May require frequent changes if saturation is reached	
	Maintains moist environment		
	Easy to apply		
NPWT	Promotes granulation	Not ideal for fragile or bleeding-prone wounds	Conditional—More suitable for complex or surgical wounds than early-stage chronicity reversal; use selectively
	Reduces edema	Requires skilled application and device management	
	Effective for large or complex wounds	Peri-wound maceration risk if not properly sealed	
	Supports exudate removal and perfusion		

Social/Economic Barriers

Psychosocial behaviors that have been shown to impede wound healing include stress, smoking and vaping [47,48], and alcohol abuse [45]. Counseling to address these habits is recommended to facilitate wound closure. Lack of or inadequate family support (including living alone), dementia, and depression are also considerations in planning effective patient and wound management. Finally, patients may not receive follow-up due to extrinsic factors, such as transportation, insurance, and ability to access care. For example, a patient who relies on a family member, friend, or neighbor to transport them to appointments will be beholden to their schedule and may not be able to attend weekly appointments. Similarly, insufficient insurance coverage or the need for prior authorization can lead to fewer and/or delayed visits. Finally, some patients live in areas with inadequate clinic access, where booking an appointment with a specialist may take longer than is ideal. When these factors are not addressed, wounds are often left to stagnate or likely worsen progressively.

How to use the BIOMES℠ tool

Low-, moderate-, and high-risk wounds are discerned based on the presence of the six BIOMES℠ factors, guiding practitioners in deciding whether advanced therapies should be implemented and when to refer to a wound specialist. Interpretation is as follows:

No BIOMES℠ barriers: low risk and no specialized care are indicated; however, the best practice principles of exudate management and moist wound healing should be followed for optimal outcomes. Should the wound fail to improve significantly within two weeks, the patient should be referred to a wound care specialist.

1 BIOMES℠ barrier: moderate risk; the risk factor needs to be addressed along with providing exudate management and moist wound healing. Refer to a wound care specialist or to a medical specialist if the risk factor is outside the evaluating clinician’s area of expertise (e.g., vascular insufficiency, autoimmune disorders, tissue infection). Should the wound fail to improve significantly within two weeks, the patient should be referred to a wound care specialist.

2+ BIOMES℠ barriers: high risk; the patient should be referred to a wound care specialist as well as to medical specialists for comorbidities.

**Figure 1:**
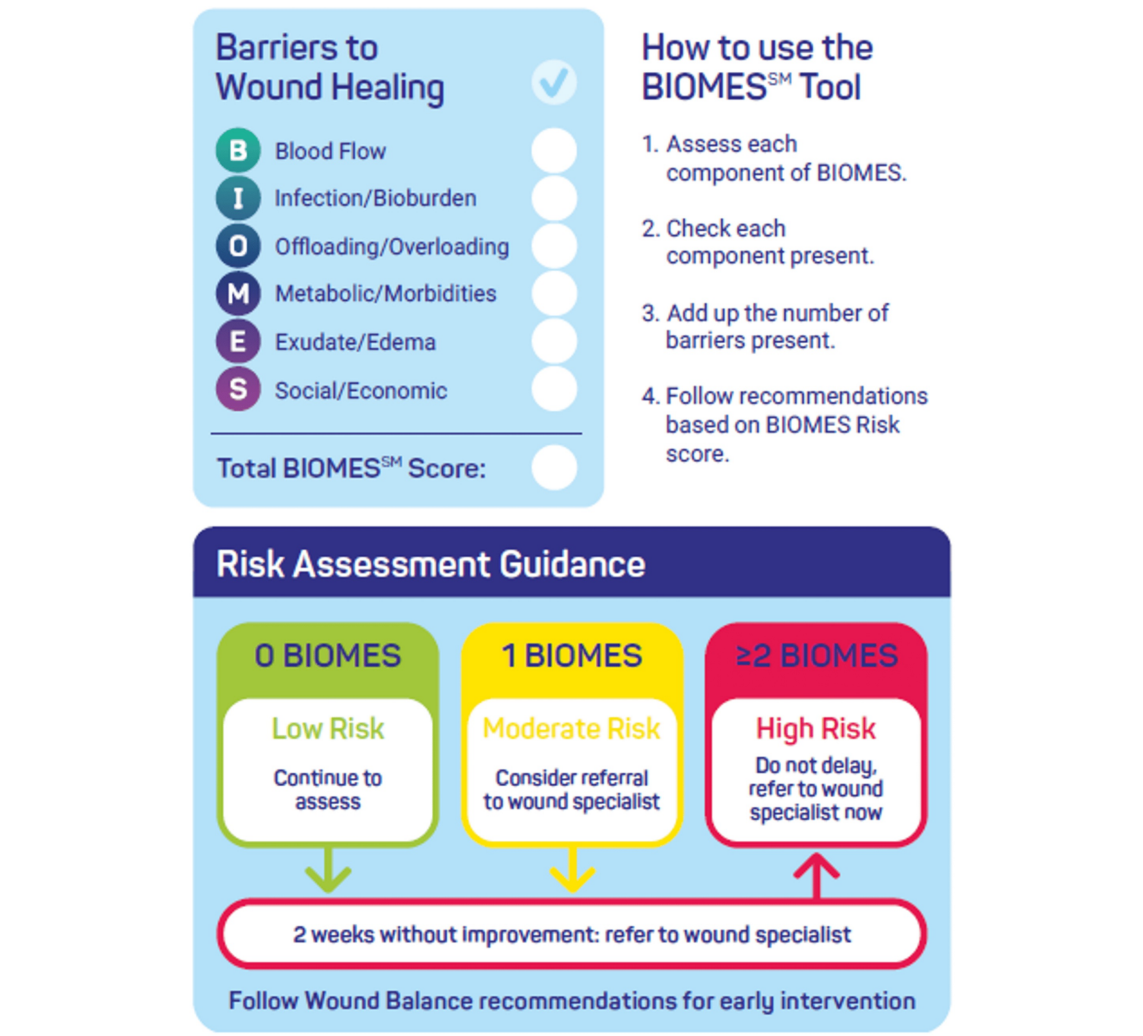
The BIOMES tool BIOMES^SM^ was created by Trent Brookshier, DPM, and is a service mark of HARTMANN USA, Inc, © 2024 HARTMANN USA, Inc. The BIOMES^SM^ tool serves as a simple guide for primary care practitioners to assess a patient’s risk and determine whether they should be referred to a specialist based on 6 key elements of wound healing.

For example, if a patient has a history of PAD or is a smoker, they would have 1 BIOMES℠ point. If the patient has diabetes, that would be another BIOMES℠ point. If the patient has difficulty with access to care or other social/economic barriers, that would be another BIOMES℠ point. If the patient has heavy exudate or difficult-to-manage moisture, that would be another point. If the patient has a hard time offloading the wound or it is on a weight-bearing surface, that will qualify as a BIOMES℠ point as well. Accumulated points are used to determine the level of risk. The tool is presented in Figure 1.

Case Study #1

**Figure 2:**
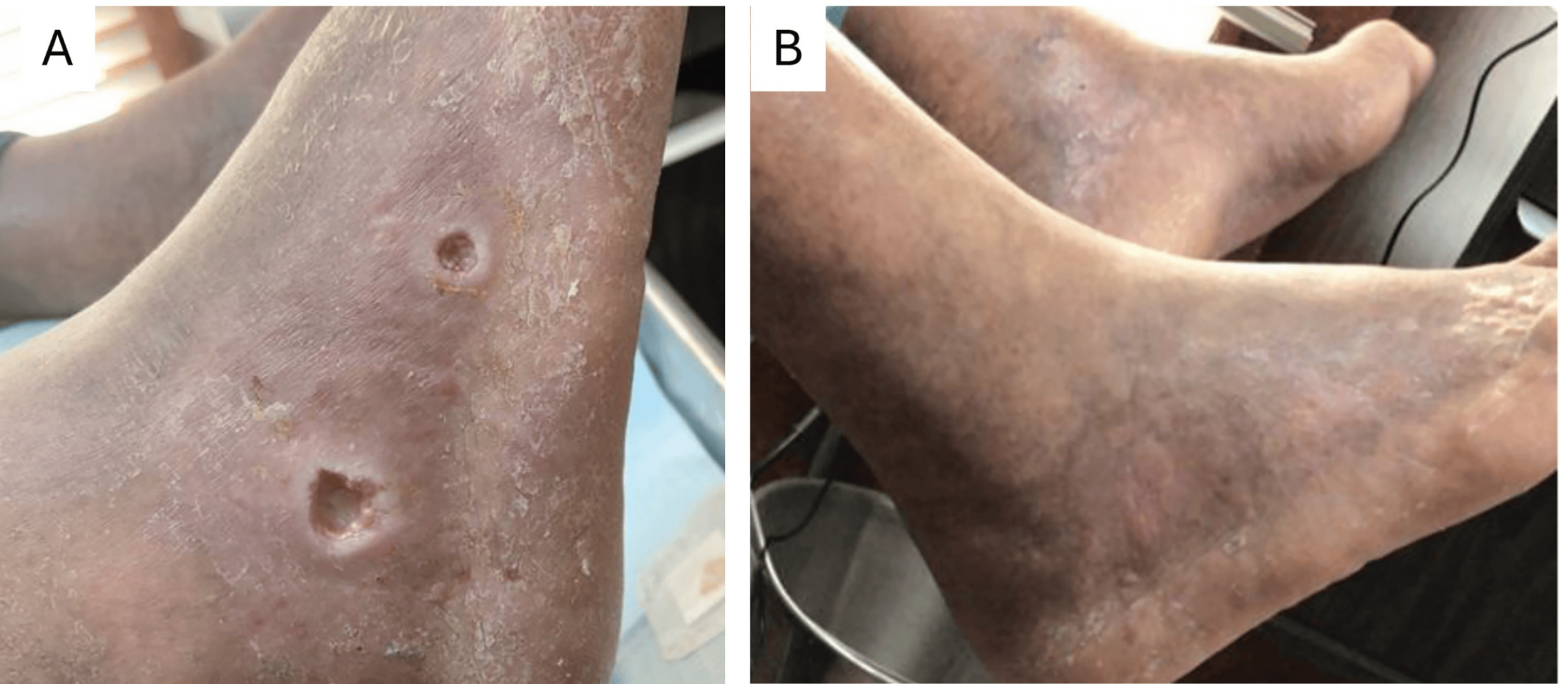
Case study #1: right foot wound before (A) and after (B) treatment by vascular specialist Case study images provided courtesy of Dr. Brookshier.

The patient is a 70-year-old male with a history of type 2 diabetes who resides in a retirement home. He has a wound on his right foot of eight months’ duration measuring 2.5 cm x 1.4 cm x 0.6 cm (Figure 1). The patient has been receiving standard wound care at a facility with no improvement. The patient was eventually referred to a wound specialist who helped assess the blood flow and metabolic issues, giving him a BIOMES℠ score of 2. The wound specialist coordinated care with a vascular specialist for procedures to improve blood flow. The wound specialist consulted with the primary care physician to improve the patient’s hemoglobin A1C. Wound care consisted of weekly debridement, application of a skin substitute (donated human allograft tissue), a multilayer silicone border SAP secondary dressing (Zetuvit® Plus Silicone Border), and a compression dressing. The wound was completely closed after six weeks (Figure 2). 

Case Study #2

**Figure 3:**
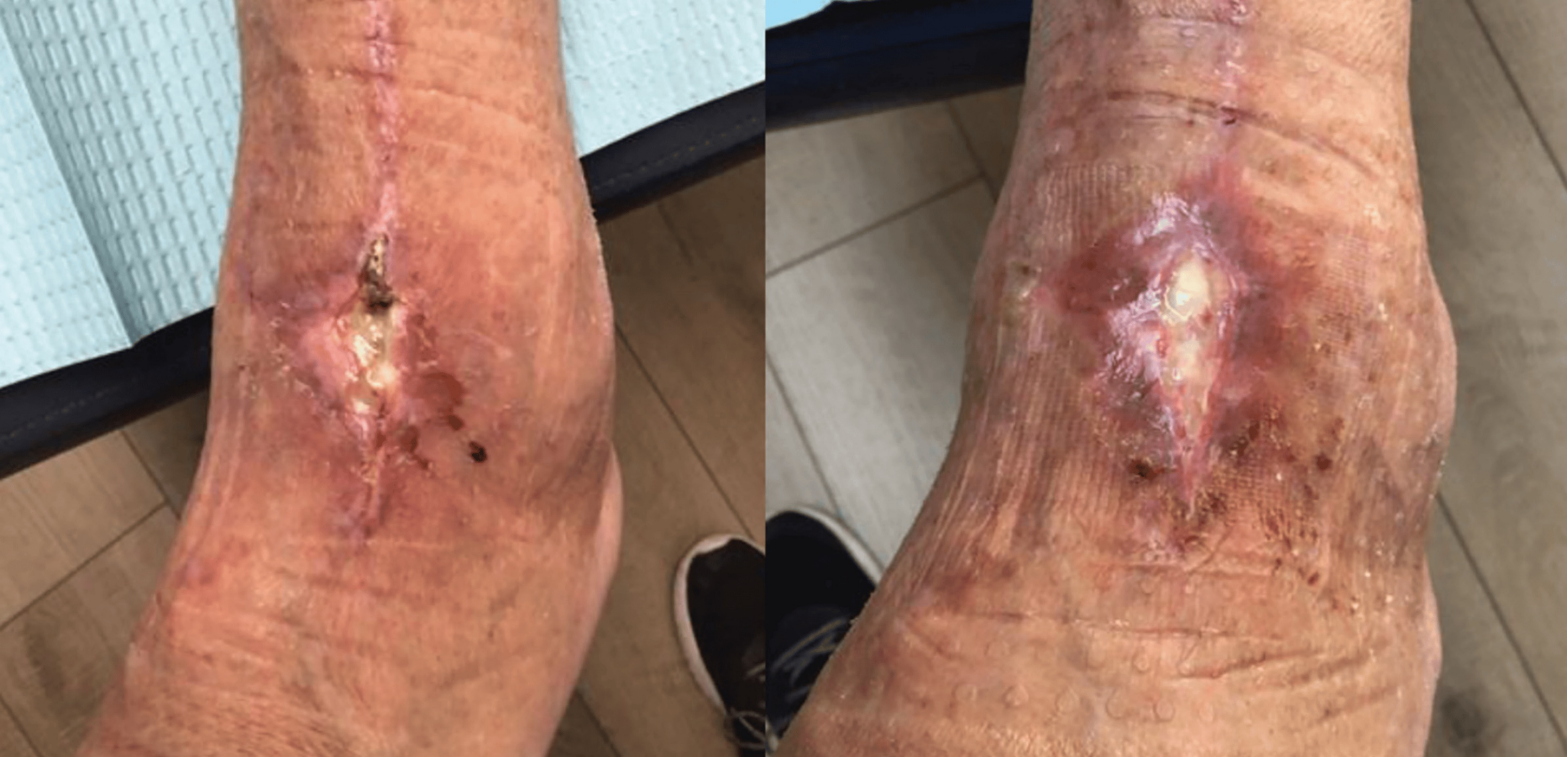
Case study #2: left ankle wound before treatment Case study images provided courtesy of Dr. Brookshier.

**Figure 4:**
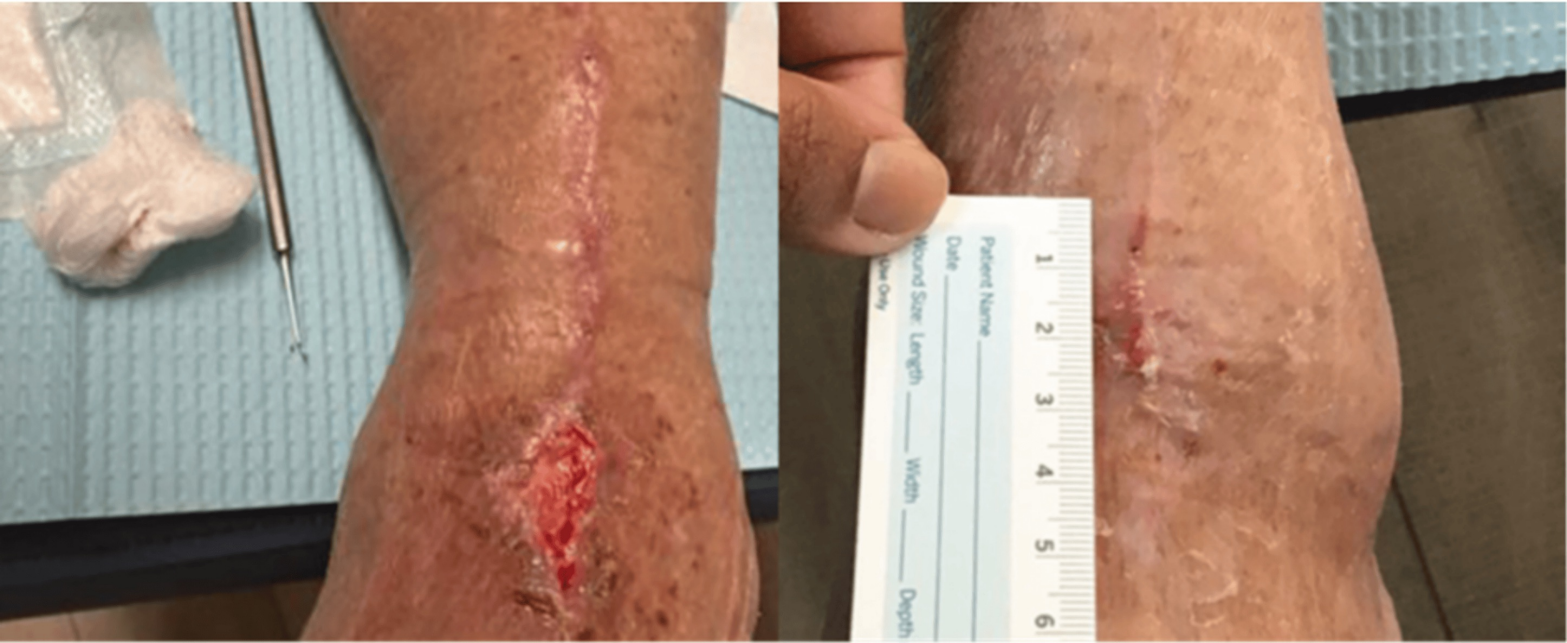
Case study #2: left ankle wound after treatment with antibiotics and dressings Case study images provided courtesy of Dr. Brookshier.

The patient is a 70-year-old female who developed wound dehiscence on a surgical incision following left total ankle replacement. The wound measured 4 cm x 3 cm x 0.2 cm and had eschar and non-viable fibrous tissue in the wound base (Figure 3). Once seen by a specialist, the assessment using the BIOMES℠ screening tool revealed the following barriers to healing: Infection and social concerns, as the patient would be returning to Mexico for the next three weeks with no access to wound care. This gave the patient a BIOMES^SM^ score of 2, i.e., high risk for not healing. The wound was cultured, and the patient was started on appropriate antibiotics. A fenestrated wound matrix was applied as a primary dressing, and a multilayer silicone border SAP dressing (Zetuvit® Plus Silicone Border) was applied as a secondary dressing. The patient was issued sufficient secondary dressings to change biweekly while on her trip. Thorough verbal and written instructions were given to the patient to help address the lack of care she would receive over the next three weeks. The wound reached full closure after five weeks of treatment (Figure 4).

Case Study #3

**Figure 5:**
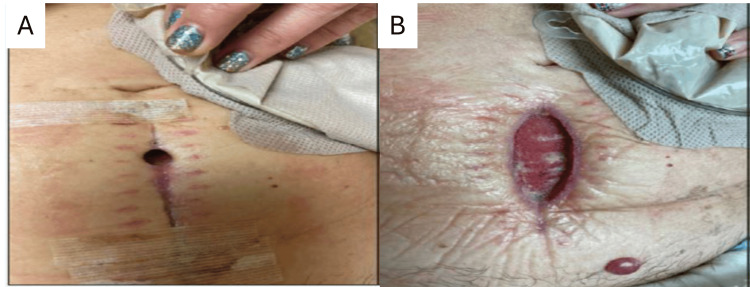
Case study #3: dehisced abdominal incision pre-(A) and post-sharp incisional debridement (B) Case study images provided courtesy of Dr. Swoboda.

**Figure 6:**
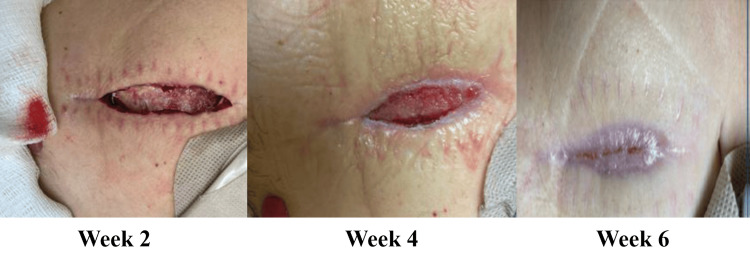
Case study #3: dehisced abdominal incision progress Case study images provided courtesy of Dr. Swoboda.

The patient is a 47-year-old female seen for a dehisced abdominal incision status post‑perforated sigmoid colon, small bowel resection, and Hartmann’s procedure, measuring 6.5 cm x 1.8 cm x 1.4 cm (Figure 5). Using the BIOMES^SM^ screening tool, the following barriers to healing were noted: infection/bioburden and metabolic/morbidities, giving the patient a BIOMES^SM^ risk score of 2. After negative pressure wound therapy, the patient was transitioned to a collagen matrix dressing with silver and EDTA (ColActive® PLUS Ag), a multilayer silicone border SAP dressing (Zetuvit® Plus Silicone Border), and began diet and lifestyle modification. The wound was fully closed in six weeks (Figure 6). 

Case Study #4

**Figure 7:**
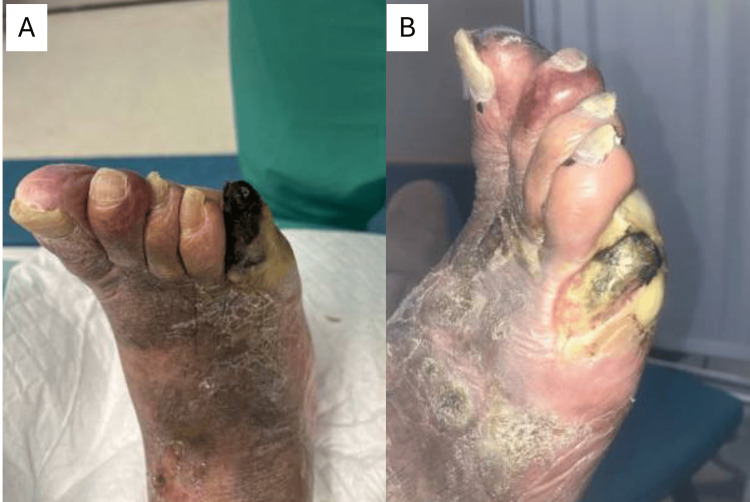
Case study #4: right foot wound at initial presentation (A) and two months later (B) Case study images provided courtesy of Dr. Brookshier.

The patient is a 72-year-old male with a history of end-stage renal disease on home dialysis, non-insulin-dependent diabetes mellitus type 2, hypertension, hyperlipidemia, anemia of chronic disease, obesity, and peripheral arterial disease. He was admitted for a right foot wound (Figure 7) and seen by emergency medicine, nephrology, and internal medicine physicians, but not by a wound specialist until two months later. Unfortunately, despite best efforts from the hospital and wound teams, the infection spread, and the patient required a foot amputation, followed by an eventual below-the-knee amputation due to microvascular complications. If the BIOMES^SM^ tool were applied in a similar case to this, the patient would have been immediately referred to a wound specialist, given the following barriers to wound healing: blood flow, offloading/overloading, and metabolic/morbidities, giving the patient a BIOMES^SM^ risk score of 3.

Case Study #5

**Figure 8:**
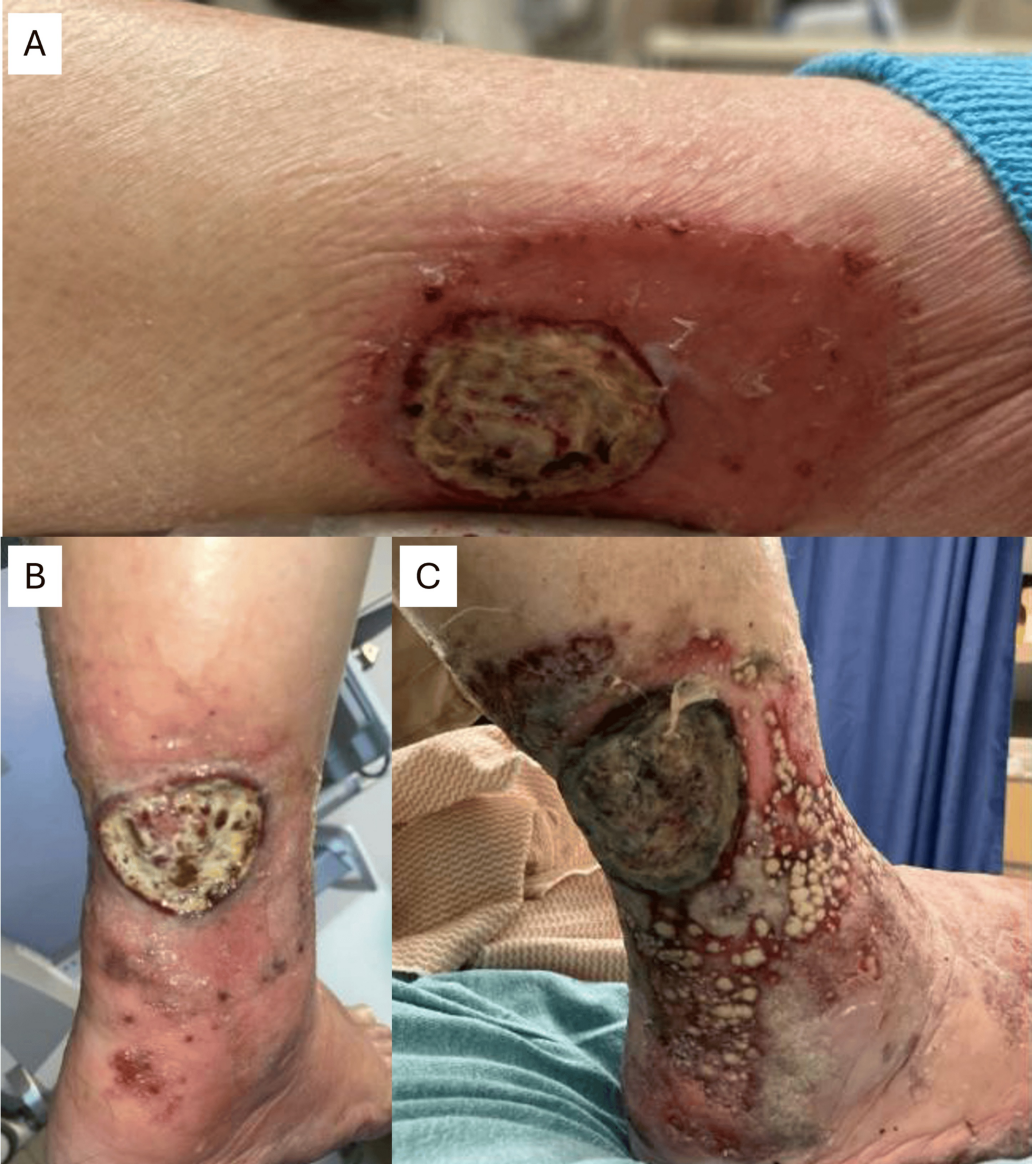
Case study #5: right leg wound at initial presentation (A, B) and three months later (C) Case study images provided courtesy of Dr. Brookshier.

The patient is a 78-year-old male with a history of hypertension, hyperlipidemia, prior smoking, coronary artery disease status post-coronary artery bypass graft, and peripheral arterial disease with bilateral lower extremity claudication. He presented with a right leg wound, which was judged to be manageable despite daily dressing changes due to odor and drainage (Figure 8), and three months later, was finally referred to a wound specialist. Unfortunately, following hospitalization, the patient became septic and eventually passed away. If the BIOMES^SM^ tool were applied in a similar case to this, the patient would have been immediately referred to a wound specialist, given the following barriers to wound healing: blood flow, exudate/edema, and metabolic/morbidity, giving the patient a BIOMES^SM^ risk score of 3.

## Discussion

The BIOMES℠ screening tool has been successfully integrated into the author’s practice, and the approach aligns seamlessly with the pillars and principles of wound care. The pillars include encompassing and understanding the disease process, wound bed preparation, addressing nutritional deficiencies, education, mental/social factors, adjunctive processes/procedures, and prevention. Adherence to these principles ensures the following components of comprehensive care: addressing hemostasis, wound classification, risk assessment, infection control, debridement, moisture control, analgesia, and wound closure.

The BIOMES℠ approach both supports and is supported by the concept of TIMERS (tissue, infection/inflammation, moisture, edge, regeneration/repair, social factors), an acronym for a best practice system that ensures appropriate and timely strategies for wound healing (Table 4). The first four components describe the steps of wound bed preparation and re-epithelialization. It should be noted that once a wound is re-epithelialized, it is considered closed; however, a wound is not healed until it completes the remodeling process to restore tensile strength of the tissue, a process that can take 6 months to 2 years, depending on the size, depth, and location of the wound. Repair and regeneration address the use of cellular, acellular, and matrix products to support cellular infiltration and stimulate cellular activity by using signal molecules or growth factors [49]. The S in TIMERS emphasizes the importance of patient engagement to increase the likelihood of wound healing and includes the social and economic barriers in the S component of the BIOMES℠ tool. TIMERS includes interventions that require experienced and capable wound specialists who are trained beyond routine interventions in general practice, family practice, or emergency medicine; thus, the use of the BIOMES℠ tool to identify moderate- to high-risk wounds is beneficial in making appropriate referrals.

**Table 4:** TIMERS for wound bed preparation and healing TIMERS: tissue, infection/inflammation, moisture, edge, regeneration/repair, social factors Adapted from Atkin L, Bucko Z, Conde Montero E, et al. Implementing TIMERS: the race against hard-to-heal wounds. J Wound Care. 2019;23:S1-S50. [49]

Components of TIMERS	
Tissue	Nonviable tissue that impedes wound healing and increases risk of infection needs to be debrided in most cases, and requires special equipment, instruments, and expertise. Exceptions to the wounds that are not usually debrided include ischemic wounds, stable eschar on pressure ulcers of nonmobile patients, and pyoderma gangrenosum until after the inflammation is adequately suppressed.
Inflammation and infection	Both need to be managed as part of a best practice care plan; inflammation by treating the underlying disorders, and infection/bioburden by antimicrobial dressings or adjunctive therapies (e.g., ultraviolet C, pulsed lavage with suction, or negative pressure therapy with instillation).
Moisture	A balanced, moist wound environment is required for healing. Advanced dressing selection or adjunctive therapy application is used to manage excessive exudate; moist dressings such as hydrogels are used to hydrate dry wounds. Superabsorbent dressings that contain polyacrylate polymers (SAPs) have a very high fluid absorption capacity (up to 100 times their own weight), and additionally have the ability to bind and sequester potential wound inhibitors (e.g., proteases such as MMP2 and elastase, or microorganisms) inside the core of the dressing, ensuring that exudate or inhibitors do not further damage the tissue and inhibit healing [50-52]. In addition, edema management is critical, especially for lower extremity wounds, and requires advanced training and expertise to select and apply the optimal compression system for each patient [53,54].
Edges	When the wound bed is clear of all nonviable tissue and is granulating, re-epithelialization occurs at the edges of the wound bed. Monitoring the edges guides treatment interventions so that epithelial migration can advance in a timely manner to obtain full closure [55,56].
Regeneration and repair of tissue	Wound closure can be encouraged by providing a matrix to support cell infiltration, stimulating cell activity, oxygen therapy, or using stem cells. Advanced therapies should be considered only after the physician has addressed risk factors for a hard-to-heal wound [49].
Social factors	Social risk factors to poor wound healing/poor adherence include both manageable and uncontrollable risk factors. Manageable risk factors, such as patient education about their wound, can be mitigated, whereas uncontrollable risk factors, such as the patient’s living conditions and presence of dementia, cannot [49].

## Conclusions

The proposed BIOMES℠ approach introduces a simple acronym that emphasizes early identification of wound risk factors, thus becoming an intervention strategy that creates a bridge between non-specialist medical teams (including general practitioners, family medicine physicians, or urgent care teams) and wound care specialists. Working together, these teams can provide comprehensive, holistic care to patients with wounds of all types, decrease healing times, reduce the cost of care, and ultimately save limbs and lives.
